# Hippocampal iron patterns in aging and mild cognitive impairment

**DOI:** 10.3389/fnagi.2025.1598859

**Published:** 2025-07-02

**Authors:** Sonja M. Kagerer, Laetitia Vionnet, Jiri M. G. van Bergen, Rafael Meyer, Anton F. Gietl, Klaas P. Pruessmann, Christoph Hock, Paul G. Unschuld

**Affiliations:** ^1^Institute for Regenerative Medicine (IREM), University of Zurich, Schlieren, Switzerland; ^2^Geriatric Psychiatry and Psychotherapy, University Hospital of Psychiatry Zurich (PUK), Zurich, Switzerland; ^3^Department of Psychiatry, University of Geneva (UniGE), Geneva, Switzerland; ^4^Institute for Biomedical Engineering, University of Zurich and ETH Zurich, Zurich, Switzerland; ^5^Neurimmune, Schlieren, Switzerland; ^6^Geriatric Psychiatry Service, University Hospitals of Geneva (HUG), Thônex, Switzerland

**Keywords:** ultra-high field MRI, 7 tesla, QSM, mild cognitive impairment, hippocampus subfields, entorhinal cortex, iron, real-time field control

## Abstract

**Introduction:**

The entorhinal cortex (EC)-hippocampus system is critical for memory and affected early in Alzheimer’s disease (AD). Cognitive dysfunction in AD is linked to neuropathological changes, including non-heme iron accumulation in vulnerable brain regions. This study characterized iron distribution in the EC-hippocampus system using ultra-high field (UHF) magnetic resonance imaging (MRI) at 7 Tesla (T) in aging and mild cognitive impairment (MCI), an AD at-risk state.

**Methods:**

40 participants (mean age [SD] 69.2 [7.42] years; 12 mild cognitive impairment (MCI), 28 cognitively healthy controls (HC)) underwent UHF MRI at 7 T with turbo spin echo and quantitative susceptibility mapping (QSM). Gray matter segmentation was performed using FreeSurfer software. Intraclass correlation coefficients (ICCs) were calculated for hippocampal and EC measures.

**Results:**

ICCs for mean susceptibilities were 0.61 overall, 0.58 for HC, and 0.69 for MCI, with significant group differences between HC and MCI (Kolmogorov–Smirnov test, *k* = 0.625, *p* ≤ 0.05).

**Discussion:**

Our findings suggest a higher coherence of non-heme iron distribution in MCI. An increasingly uniform distribution of iron in MCI could reflect a clinical continuum ranging from healthy aging to pathologic brain change and cognitive disorder. This highlights the potential of non-heme iron as a biomarker for early AD co-pathology.

## 1 Introduction

The entorhinal cortex (EC)-hippocampus system closely interacts with the neocortex and plays a central role in memory processing (Duvernoy and Risold, 2005; Rao et al., 2022). Aging related cognitive disorders such as Alzheimer’s disease (AD) have been shown to particularly affect functionality of the EC-hippocampus system (Khan et al., 2014; Rao et al., 2022). While Alzheimer’s disease is characterized by early alteration of core biomarkers indicating progressive accumulation of amyloid beta (Aβ), it is the characteristic progression of tauopathy which is linked to emergence of the clinical syndrome (Bejanin et al., 2017). In addition, manifestation and severity of the cognitive syndrome in AD is closely linked to presence of non-specific AD-comorbidity (Jack et al., 2024). Mild cognitive impairment (MCI) is an at-risk state for the development of AD, especially when episodic memory is impaired.

Accumulation of non-heme iron in vulnerable brain regions has been shown to be closely associated with AD pathology (Ayton et al., 2020; Ravanfar et al., 2021). Interestingly, cerebral iron deposition correlates well with Braak stages and is closely associated with the progression of cognitive decline (Ayton et al., 2017b; van Duijn et al., 2017; Ayton et al., 2020). An association of cerebral iron burden with cognitive decline or impaired cognitive functionality has also been found for persons with MCI and persons at risk for AD that still showed normal cognitive function (Ayton et al., 2015; Ayton et al., 2017a; Kagerer et al., 2020).

Validity of quantitative susceptibility mapping (QSM) MRI for measuring non-heme iron the human brain has been demonstrated by several studies (Langkammer et al., 2012; Sun et al., 2015; Yao et al., 2023). During aging, iron has been demonstrated to accumulate in cortical and deep gray matter structures (Hallgren and Sourander, 1958; Acosta-Cabronero et al., 2016). In AD patients, elevated iron deposition as estimated with QSM have been reported in the frontal, temporal, parietal and occipital cortex, hippocampus, and deep brain nuclei compared to healthy controls (HC) (Ravanfar et al., 2021). For MCI patients, increased iron deposition has been observed in the hippocampus, precuneus, cingulate and EC (van Bergen et al., 2016; Kim et al., 2017). So far there is only few published information on iron accumulation in hippocampal subfields and the EC: Zeineh and colleagues observed iron-containing microglia primarily in the subiculum using ex-vivo MRI and histological staining of AD-brains (Zeineh et al., 2015). A recent QSM study reported elevated iron in hippocampal fimbria of persons with AD (Au et al., 2021).

Advances in ultra-high field (UHF) magnetic resonance imaging (MRI) at 7 Tesla (T) allow for greater spatial resolution and precise segmentation of hippocampal subfields (de Flores et al., 2015; Kagerer et al., 2022; Yao et al., 2023; Stirnberg et al., 2024). Combination with real-time field control has been demonstrated to further stabilize and improve image quality (Duerst et al., 2015; Duerst et al., 2016; Wyss et al., 2017; Vionnet et al., 2021).

In the current study we used a sophisticated UHF MRI at 7 T protocol combined with real-time field control to characterize iron distribution in the EC and hippocampal subfields at high precision.

To our knowledge, this is the first *in vivo* assessment of iron distribution in structures of the EC-hippocampal system, as well as its redistribution as a possible correlate of age-related pathology and subsequent cognitive impairment.

## 2 Materials and methods

### 2.1 Participants

All participants for the current study were recruited in the cantone of Zurich, Switzerland, from ongoing longitudinal studies at our center. Written informed consent was obtained from all participants before inclusion in the study. All study procedures were conducted in accordance with local regulatory requirements, the Declaration of Helsinki (World Medical Association, 1991) and approved by the cantonal ethics committee of Zurich, Switzerland.

Inclusion criteria were age equal or above 50 years and German language proficiency.

Exclusion criteria were diagnosis of dementia, presence of any condition possibly affecting cognition or study participation (e.g., severe hearing loss), any present medication that may affect cognition, present or past substance abuse, any serious medical or psychiatric illness, general MRI exclusion criteria or significant exposure to radiation. Furthermore, participants that showed any evidence of infarction or inflammation in the cranial MRI were not included.

All subjects completed a cranial UHF MRI at 7 T and received a comprehensive neurological and psychiatric examination as well as a complete neuropsychological workup.

### 2.2 Cognitive assessment

In order to thoroughly assess the cognitive function, all study participants completed a standardized neuropsychological test battery including the Mini Mental State Examination (MMSE) (Folstein, Folstein et al., 1975), the Revised Boston Naming Test (BNT) (Nicholas et al., 1988), Digit Spans Backward (Gregoire, 1997), Trail Making Test (TMT) B/A (Tombaugh, 2004) and the Verbal Learning and Memory Test (VLMT): delayed recall (Lange et al., 2002). Participants were categorized as HC or MCI according to established criteria for MCI (Albert et al., 2011).

### 2.3 Acquisition of MRI data

MRI acquisition of all study participants was carried out on the same 7 T Achieva whole-body scanner (Philips Healthcare, Best, The Netherlands) equipped with a Nova Medical quadrature transmit head coil and 32-channel receive coil array (Nova Medical, Wilmington, DE, USA), located at the Institute for Biomedical Engineering (IBT) at the Swiss Federal Institute of Technology at Zurich, Switzerland (ETH Zurich). The identical real-time field control setup and protocol was used for all participants.

The protocol included a whole brain 3D magnetization-prepared 2 rapid acquisition gradient echo (MP2RAGE) scan (voxel size = 0.8 × 0.8 × 0.8 mm^3^; FOV = 200 × 200 × 144 mm^3^; TR / TE / TIs / flip angle = 6.9 ms /2.7 ms /131 ms and 1897 ms / 5°; scan duration = 6:53 min), that was reconstructed and used to plan a high-resolution coronal turbo spin echo (TSE) scan with a field of view covering the hippocampus (voxel size = 0.25 × 0.25 × 1.2 mm^3^; FOV = 200 × 200 × 144 mm^3^; TR/TE/flip angle = 6019.3 ms /80 ms/5°; scan duration = 8:38 min).

For the calculation of QSM a high-resolution 3D GRE T2*-weighted structural scan of the whole brain was performed (voxel size = 0.6 × 0.6 × 2 mm^3^; FOV = 200 × 180.72 × 130.2 mm^3^; TR/single TE/flip angle = 40 ms/ 14 ms/10°; scan duration = 12:34 min).

### 2.4 Real-time field control

**Figure 1:**
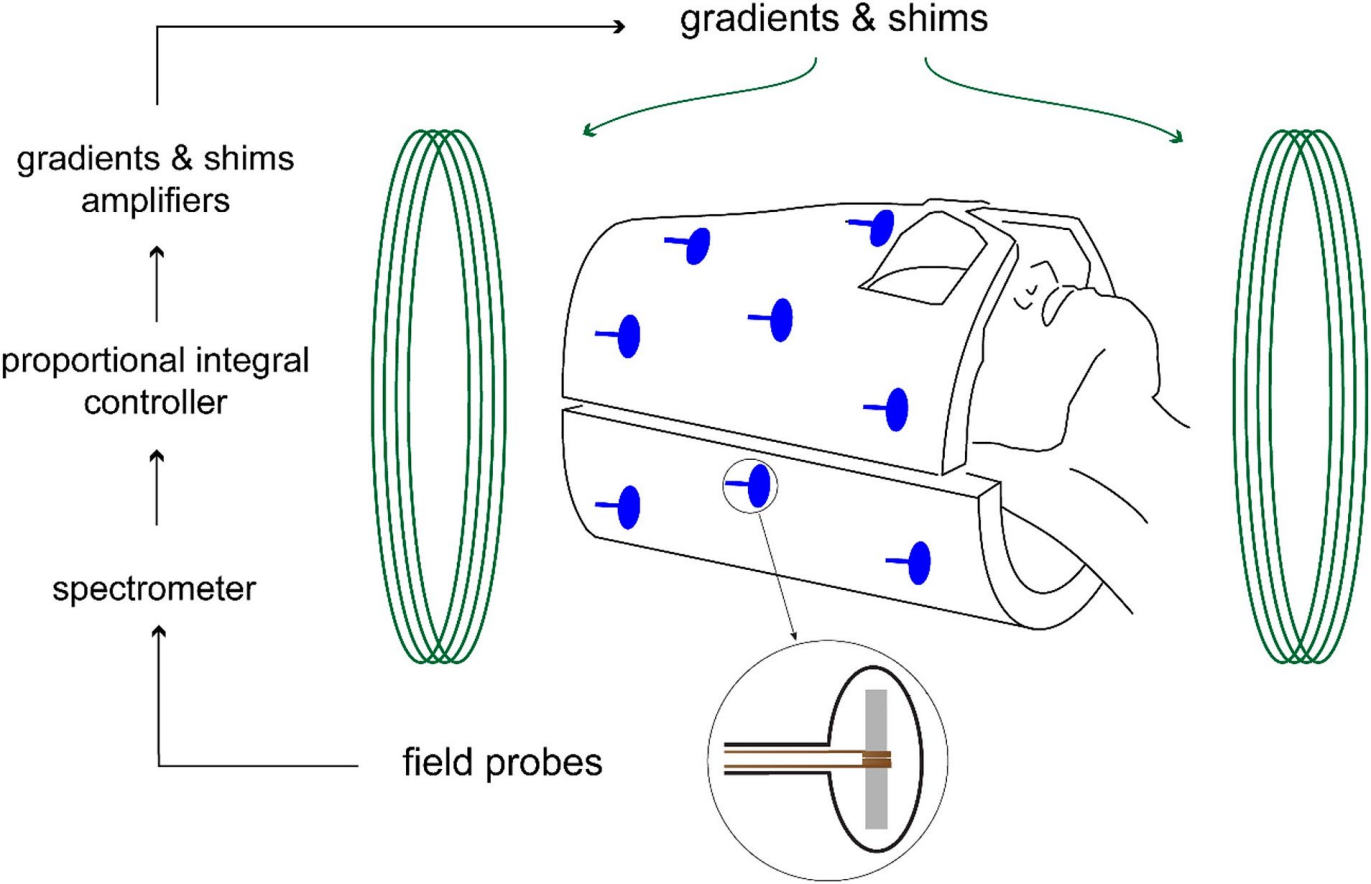
Field control set-up. 16 Fluorine field probes measure the field every 120 ms. After processing by the spectrometer, the controller estimates the required shim inputs that maintains the field to the target and actuates the gradients and shims amplifiers.

**Figure 2:**
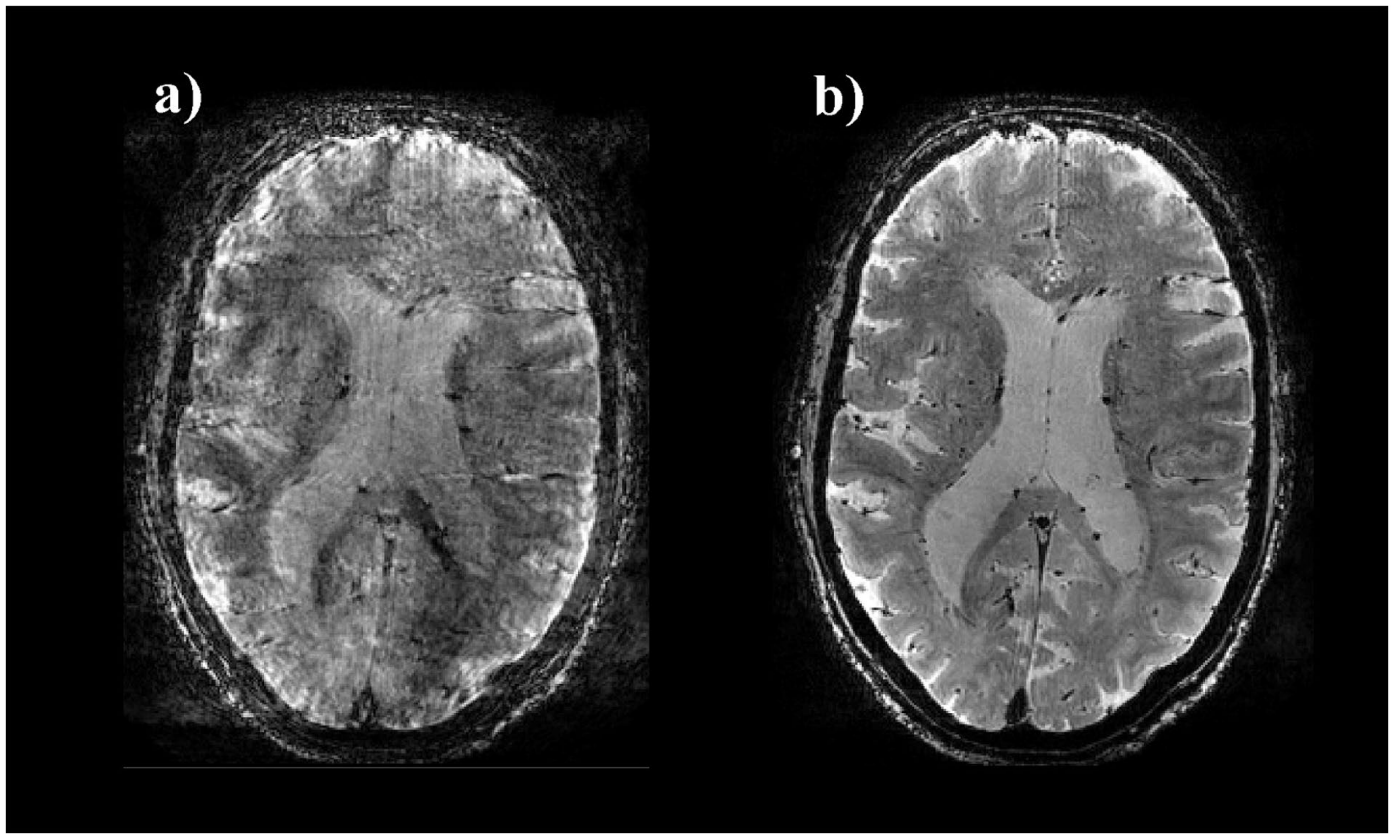
Image quality of 3D GRE T2* images with and without field control. Axial view of 3D GRE T2* images of one CN subject without **(a)** and with **(b)** real-time field control: typical artifacts due to field fluctuations are removed in **(b)**.

Real-time field control was applied during the 3D GRE sequences, as previously described by our group (Duerst et al., 2015; Duerst et al., 2016; Wyss et al., 2017; Vionnet et al., 2021). This technique improves image quality by mitigating physiologically induced field inhomogeneities to which GRE sequences are particularly sensitive especially at ultra-high field strengths (van Gelderen et al., 2007; Duerst et al., 2015; Duerst et al., 2016). Field sensing was performed using 16 fluorine-based transmit/receive nuclear magnetic resonance (NMR) field probes arranged cylindrically around the head receive array. In a calibration step, reference field values were measured at the sensor positions for each shim field pattern prior to image acquisition. During the scan, field values were acquired for every second slice TR, after the read-out and 7 ms prior to the next excitation. The field-probe signal was acquired for 3.1 ms and data included between 0 and 3 ms was processed on a separate stand-alone spectrometer to compute the field values by derivation as described earlier by our group (De Zanche et al., 2008; Dietrich et al., 2016). A proportional-integral controller computed the field corrections, and the corresponding field actuation was performed by real-time 3rd-order shim adjustment. The setup is illustrated in Figure 1, improvement in image quality is illustrated in Figure 2.

### 2.5 MRI data reconstruction for QSM

QSM images were calculated from masked 3D T2*- weighted data phase images using the total-generalized-variation (TGV) method as proposed by Langkammer and colleagues (Langkammer et al., 2015). This single-step approach integrates Laplacian-based phase unwrapping, background field removal and dipole inversion reducing error propagation. The brain mask was generated using FSL’s brain extraction tool (BET, FMRIB Oxford, UK) using a fractional intensity threshold of 0.1. TGV reconstruction parameters followed Langkammer et al.’s protocol (*α* = (0.0015, 0.0005); 1,000 iterations) (Langkammer et al., 2015). Spatially confined vascular objects such as veins and microbleeds were excluded from estimation of regional susceptibility values.

### 2.6 Gray matter segmentation

Hippocampal subfield segmentation was performed using the “mri_robust_register” function implemented in FreeSurfer Version 6.0^1^ (Iglesias, Augustinack et al., 2015; Worker et al., 2018). This particular FreeSurfer software version has been developed using 7 T data, particularly from the hippocampal subfields, and has been validated for the use of 7 T (Iglesias et al., 2015; Zaretskaya et al., 2018). Preprocessing was conducted via an in-house pipeline (Kagerer et al., 2022). MP2RAGE and TSE structural scans were used as inputs to the segmentation pipeline. Pre-processing comprised conversion from three-dimensional Nifti format, motion correction, averaging of multiple high-resolution images, transformation into Talairach space, intensity normalization, brain extraction, segmentation of subcortical structures, tessellation of the gray-white matter boundary, automated topology correction, and surface deformation to refine the gray/white matter and gray matter/cerebrospinal fluid boundaries. The left and right hippocampus were segmented each into their subfields. For further analysis, subfields with established relevance for cognitive processes were chosen: parasubiculum, presubiculum, subiculum, cornu ammonis (CA)1, CA3, CA4 and granule cell layer of dentate gyrus (GCDG). The entorhinal cortex was also delineated. Segmentation quality was manually controlled for every participant. Lateral ventricle volume was extracted using aseg segmentation to serve as a reference for susceptibility values (Fischl et al., 2002).

### 2.7 Regional susceptibility value computation

To compute regional susceptibility, MP2RAGE images were co-registered to the GRE magnitude images to align anatomical and susceptibility data spatially. Hippocampal subfield masks derived from FreeSurfer segmentation pipeline were then applied to the co-registered QSM maps. Susceptibility values were then extracted for all segmented hippocampal subfields and the entorhinal cortex. Among several suitable reference regions—including white matter tracts and central cerebrospinal fluid areas—the region with the lowest standard deviation in mean susceptibility across all subjects was selected. In our sample, the frontal central cerebrospinal fluid region within the lateral ventricles showed the most stable signal and was therefore used as the reference for final susceptibility quantification. The lateral ventricle volume was extracted using aseg segmentation (Fischl et al., 2002). The extracted lateral ventricle volume was first eroded before calculation of the mean to avoid inclusion of structures in the surrounding of the ventricles in the reference mean susceptibility value. The calculation of the subfield’s average susceptibility was completed by dividing each subfield’s mean susceptibility value with the mean susceptibility value of the reference region as described earlier by our group (van Bergen et al., 2016).

### 2.8 Data analysis and statistics

The ICC(2,1) model (two-way random effects, single measurement) was used to assess the coherence of susceptibility distribution patterns across hippocampal subfields from mean left–right QSM values within each group. This approach allows estimation of the consistency in regional susceptibility values across individuals, providing a measure of spatial distribution uniformity (Shrout and Fleiss, 1979; McGraw and Wong, 1996).

Group differences were assessed using two-sample *t*-tests, with effect sizes estimated via Cohen’s *d*. A combined approach of principle component analysis and two-sample Kolmogorov–Smirnov test was performed to test for a significant difference of ICC coefficients between subgroups, including possible effects of covariates such as age and sex.

Data analysis was performed using MATLAB (Version 2022a, Statistics and Machine Learning Toolbox Version 12.3, Bioinformatics Toolbox Version 4.16) software.

## 3 Results

### 3.1 Study sample

The study sample included 40 old-aged participants (mean age [SD] 69.2 [7.42] years, range 57–96 years, 16 females; Table 1). Of these 28 participants were classified as HC (mean age [SD] 67.2 [6.11] years, range 57–79 years; 13 females) and 12 as MCI (mean age [SD] 73.9 [8.30] years, range 61–96 years; 3 females).

**Table 1:** Demographic and descriptive data.

	All	MCI	HC	*t*-test (p)
*N*	40	12	28	
Age	69.2 (7.42)	73.9 (8.30)	67.2 (6.11)	0.02*
Sex (females/males)	16/24	3/9	13/15	0.21
Years of education	15.9 (2.89)	16.6 (2.64)	15.6 (2.96)	0.31
MMSE	29.4 (0.78)	29.2 (0.94)	29.5 (0.69)	0.31
VLMT delayed recall	8.1 (4.28)	4.0 (3.16)	9.8 (3.44)	<0.001*

Means with standard deviation. *p < 0.05.

MCI, Mild cognitive impairment, HC, Healthy control, MMSE, Mini-mental state examination; VLMT, Verbal learning and memory test.

There were no significant differences in sex distribution, years of education or MMSE between the MCI and HC groups (*p* = 0.21), but a significant difference in age (*p* = 0.021) and VLMT delayed recall performance (*p* < 0.001).

### 3.2 Hippocampal subfield segmentation and regional volumes

**Figure 3:**
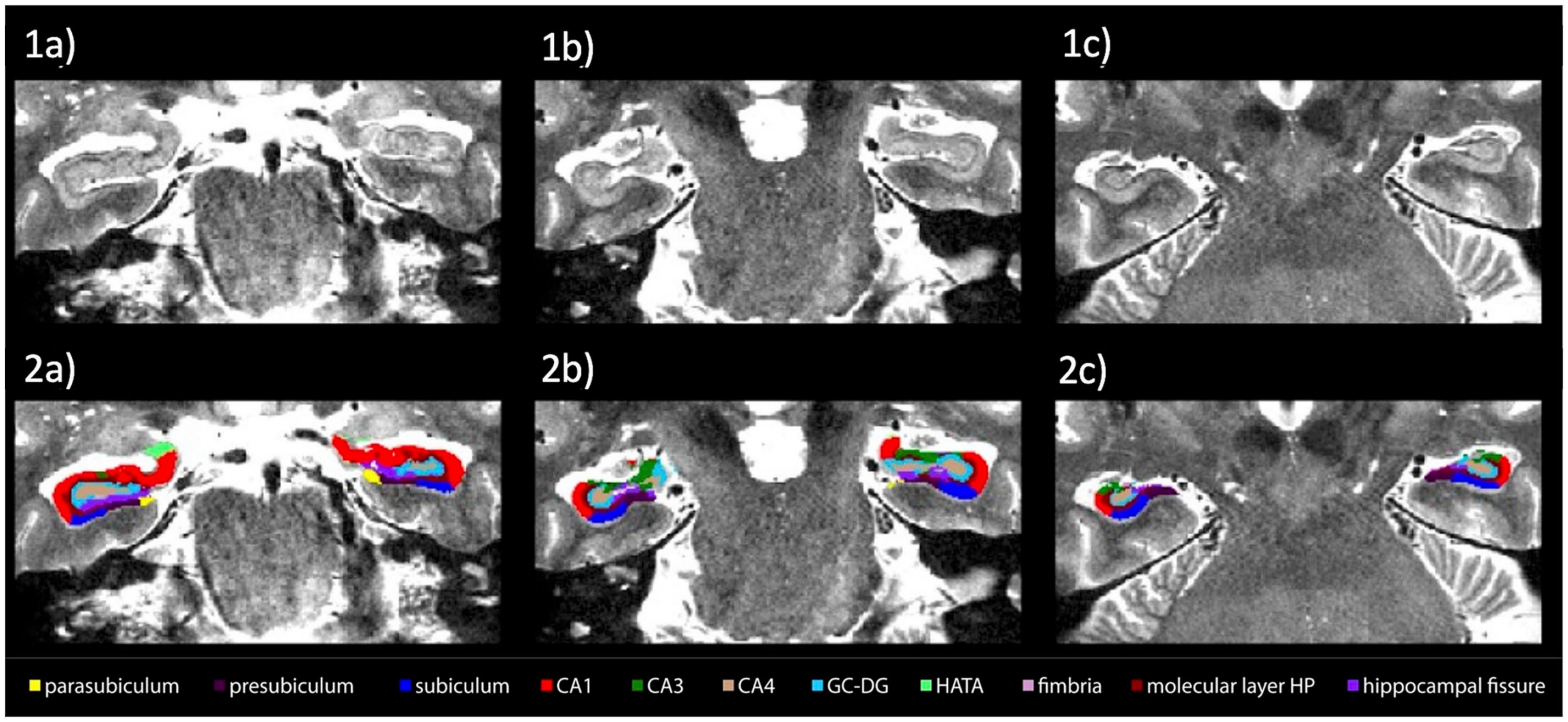
Spatial definition of hippocampal subfields by 7 Tesla high-resolution coronal turbo spin echo (TSE) sequences. Coronal view of left and right hippocampal regions of high resolution 7 Tesla TSE images of one HC **(1a–1c)** with overlay of subfield segmentation by using FreeSurfer V6.0 **(2a–2c)**. Slices are depicted from left to right from anterior to posterior.

Automatic segmentation of hippocampal subfields from both hemispheres was successfully achieved for all subjects. The accuracy of automatic subfield segmentation achieved by FreeSurfer 6.0 closely aligned with the tissue boundaries of hippocampal subfields, as discernible in high field strength 7 T T1 images. Jarque-Bera test confirmed normal distribution of subfield data (all *p* > 0.1), indicating no significant over-or undersegmentation of any hippocampal subregion. Standard deviations of subfield volumes for all participants ranged from 11.2 to 20.3% with a mean of 15.9%. Figure 3 shows coronal sections of TSE images along the hippocampus for one HC subject.

Compared to HC persons MCI patients showed significantly lower volumes of subiculum, CA1, CA4 and GCDG (*p* = 0.01, *p* = 0.02, *p* = 0.02, *p* = 0.01, Cohen’s *d* = 0.82, 0.83, 0.70 and 0.78, respectively). Results were robust to correction for multiple testing using Benjamini-Hochberg procedure (Table 2).

**Table 2:** Volumes of hippocampal subfields in mm^3^, means of left and right each with standard deviation.

Subfield	All	MCI	HC	*t*-test (p)	p FDR	Cohen’s d
Parasubiculum	47.2 (9.6)	48.6 (11.7)	46.6 (8.7)	0.59	0.67	−0.21
Presubiculum	246.9 (36.7)	231.8 (30.8)	253.3 (37.7)	0.07	0.11	0.59
Subiculum	350.1 (48.3)	323.3 (37.8)	361.6 (48.3)	0.01*	0.04*	0.82
CA1	577.4 (64.8)	541.4 (58.4)	592.9 (62.0)	0.02*	0.04*	0.83
CA3	180.6 (34.1)	171.2 (28.9)	184.6 (35.8)	0.23	0.30	0.39
CA4	216.4 (30.9)	201.7 (20.5)	222.8 (32.7)	0.02*	0.04*	0.70
GCDG	247.8 (36.6)	228.5 (26.4)	256.1 (37.6)	0.01*	0.04*	0.78
EC	1649.6 (312.6)	1665.8 (429.5)	1642.6 (256.5)	0.86	0.86	−0.07

*p < 0.05.

### 3.3 Extraction of regional susceptibility and iron load

Average susceptibility values for each hippocampal subfield, referenced to the lateral ventricle, were extracted to estimate iron load (Table 3). MCI patients showed higher iron load values in Subiculum (*p* = 0.02, Cohen’s *d* = 0.79), CA1 (*p* = 0.03, Cohen’s *d* = 0.77) and Presubiculum (*p* = 0.01, Cohen’s *d* = 0.84) compared to HC participants. However, after correction for multiple testing only a trend remained for these three subfields (*p* = 0.07).

**Table 3:** QSM values of hippocampal subfields in ppm, means of left and right each with standard deviation.

Subfield	All	MCI	HC	*t*-test (p)	p FDR	Cohen’s d
Parasubiculum	0.004 (0.008)	0.004 (0.008)	0.004 (0.009)	0.87	0.91	0.05
Presubiculum	−0.003 (0.004)	−0.0005 (0.003)	−0.004 (0.004)	0.01*	0.07	0.84
Subiculum	−0.004 (0.006)	−0.001 (0.005)	−0.006 (0.005)	0.02*	0.07	0.79
CA1	−0.004 (0.005)	−0.001 (0.005)	−0.005 (0.005)	0.03*	0.07	0.77
CA3	−0.002 (0.007)	0.001 (0.007)	−0.003 (0.007)	0.08	0.17	0.60
CA4	−0.018 (0.005)	−0.018 (0.004)	−0.018 (0.005)	0.82	0.91	0.07
GCDG	−0.013 (0.004)	−0.013 (0.003)	−0.012 (0.005)	0.91	0.91	−0.04
EC	−0.003 (0.003)	−0.002 (0.003)	−0.004 (0.003)	0.19	0.30	0.47

*p < 0.05.

### 3.4 Consistency of regional susceptibility variance across groups

**Figure 4:**
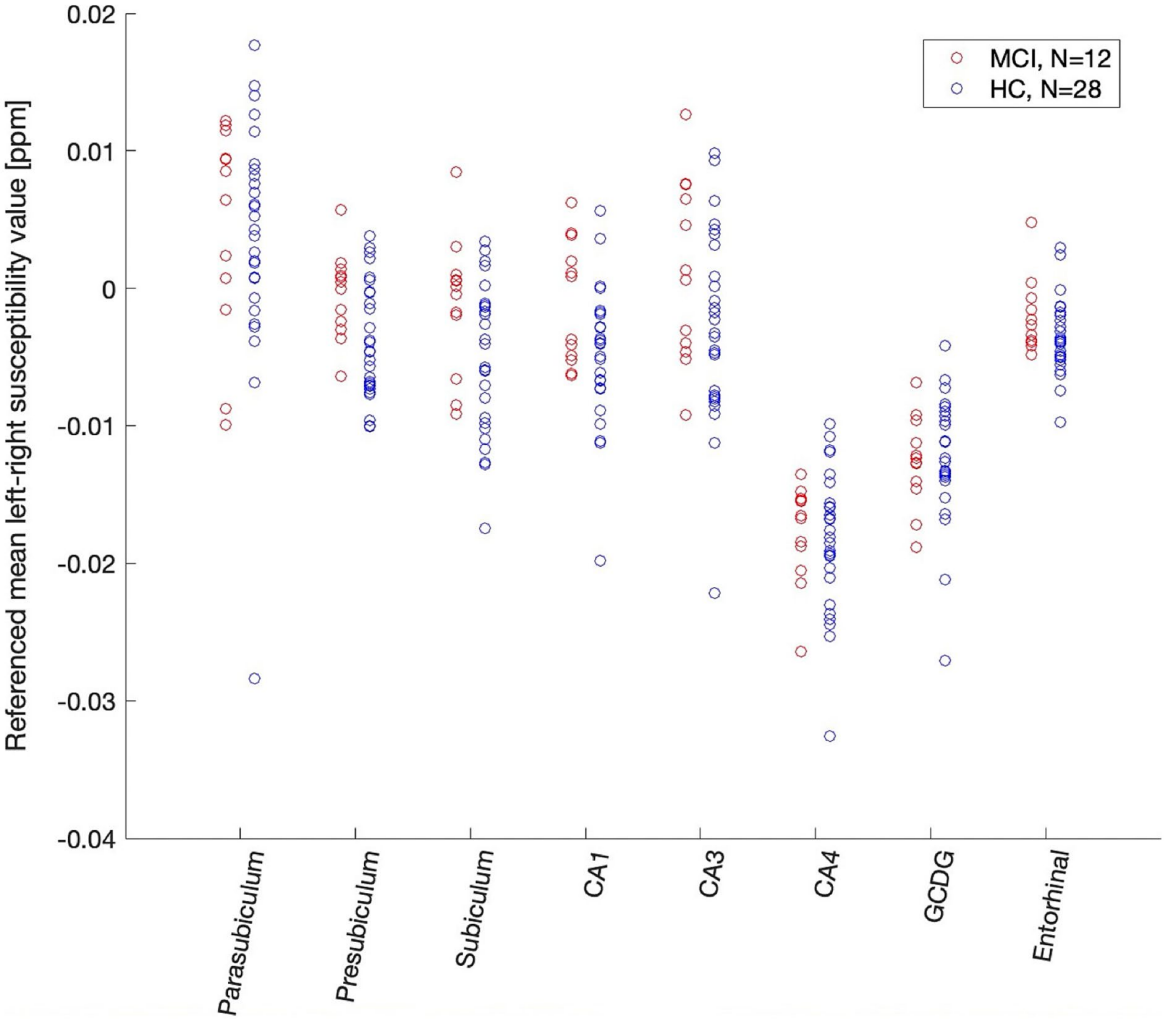
Susceptibility distribution pattern of QSM values (ppm) in all subfields in HC and MCI participants. Scatterplot of QSM values (ppm) for all participants and all analyzed hippocampal subfields and the entorhinal cortex. Intraclass correlation coefficients were high for all (0.61), HC (0.58) and MCI (0.69) participants. Two-sample Kolmogorov–Smirnov test showed a significant difference between MCI and HC participants iron distribution (*p* = 0.049). CA, cornu ammonis; GCDG, granule cell layer of dentate gyrus.

Mean regional susceptibility values for all subjects are depicted in Figure 4. The figure visualizes a high degree of consistency in the distribution of subfield susceptibility among all subjects and the subgroups. ICCs for the mean regional susceptibilities were 0.61 for all subjects (n = 40), 0.58 for the HC subjects only (*n* = 28), and 0.69 for the MCI subjects only (*n* = 12). All three coefficients are above 0.5, indicating a high consistency in the distribution of susceptibility across subfields for all groups.

For covariate control, ICCs were also calculated for a high versus low age subgroup as defined by a median split and for a female versus male subgroup (ICC high age = 0.58, ICC low age = 0.66, ICC males = 0.58, ICC females = 0.70).

### 3.5 Increased coherence of susceptibility variance in MCI

Two-sample Kolmogorov–Smirnov test revealed a significant difference in the ICCs between the HC and MCI subgroup (*k* = 0.625, *p* = 0.0497), indicating a higher coherence of regional susceptibility variance in MCI patients compared to HC participants. Cohen’s *d* indicated medium to strong effects for MCI vs. HC (Cohen’s *d* = 0.72).

Regarding confounding variables, no significant difference was found between the high vs. low age group (*k* = 0.50, *p* = 0.19), but for the female vs. male group (*k* = 0.625, *p* = 0.0497). Cohen’s d indicated small to medium effect sizes for the effect of sex (Cohen’s *d* = 0.42).

## 4 Discussion

By applying an innovative real-time field-controlled UHF MRI at 7 T setup, we found significantly higher coherence of non-heme iron patterns in the EC-hippocampus system of persons with MCI, compared to HC. While consistent patterns of iron distribution were also observable in HC, our findings might represent a continuum ranging from healthy aging to pathologic brain change and cognitive disorder. As such, higher ICC values reflect a greater consistency of susceptibility profiles across hippocampal regions within subjects, which may indicate a loss of anatomical differentiation in iron distribution as a potential marker of neurodegeneration in MCI, an AD at-risk state. To our knowledge, this is the first observation of regional distinct iron distribution patterns within the EC-hippocampal system of old aged persons.

As brain-iron deposition has been shown to be associated with progressive tauopathy in AD (Spotorno et al., 2020), further longitudinal studies are needed to confirm whether brain iron distribution follows a characteristic pattern, as it is known for tau (Braak and Braak, 1991; Bejanin et al., 2017). In this context, distinct patterns of iron distribution could potentially emerge as biomarkers of non-specific AD co-pathology, as suggested recently to play a central role for staging of AD (Jack et al., 2024). This notion might be supported by our observation of a higher coherence of hippocampal iron distribution patterns in MCI.

Our findings align well with previous studies that have demonstrated the utility of QSM iron imaging for investigating AD and brain aging (Ayton et al., 2017b; Ayton et al., 2020; Sato et al., 2022; Zhou et al., 2024). While the state of MCI in general encompasses a heterogenous group of underlying pathologies, our findings suggest that alterations in iron distribution may be found in MCI participants at increased risk for AD, as indicated by their older age, lower VLMT delayed recall performance and lower hippocampal subfield volumes compared to HC (Kagerer et al., 2022; Zhang et al., 2023). Thus, the MCI group in this study represents a population with higher risk for AD-conversion compared to general MCI samples, potentially explaining the pronounced iron distribution patterns observed.

Moreover, we found a higher coherence of non-heme iron patterns in the EC-hippocampus system in female compared to male participants. Although the effect size for sex differences was considerably smaller than for MCI versus HC, we cannot rule out an effect of sex, based on our data. This finding might align with the well-established sex-dependent AD risk that could be associated with a reduced anatomical differentiation in iron distribution. Yet, this interpretation should be made with caution as the number of female participants in the MCI group was limited, underscoring the need for confirmation in future studies.

Additionally, we report for the first time distinct differences in iron burden of Subiculum, CA1 and Presubiculum between MCI and HC participants. Although this finding remained only a trend after correcting for multiple testing - likely due to an insufficient power because of the small sample size – our results are consistent with previous research linking increased hippocampal iron to AD and not healthy aging (Acosta-Cabronero et al., 2016; Ayton et al., 2017b; Spence et al., 2022; Zhou et al., 2024).

The observation that general iron reduction in MCI and early AD does not lead to an improvement of cognitive dysfunction (Ayton et al., 2024) may support the notion that the underlying pathophysiological process in Alzheimer’s disease rather is a pathological shift of local iron than an overall increase in iron load. Given that iron is an essential metabolic resource, further studies are needed to clarify which local neuronal and cognitive processes might be compromised by this redistribution.

While our findings might help to better understand cognitive decline in disorders such as AD, they might also reflect underlying pathological processes relevant for staging of the cognitive disorder (Jack et al., 2024). As such, iron could promote cognitive dysfunction by oxidative damage and ferroptosis of neuronal tissue (Ayton et al., 2020; Gleason and Bush, 2021; Levi et al., 2024). Moreover, brain iron accumulation has been demonstrated to be implemented in neuroinflammatory pathology in AD (Zeineh et al., 2015).

For this study UHF MRI at 7 T was used, which has been demonstrated to result in significantly improved image-quality when used for susceptibility related imaging sequences such as QSM (Isaacs et al., 2021; Stirnberg et al., 2024). Ultra-high field strength leads to increased signal-to-noise ratio, higher spatial resolution and improved phase contrast due to greater sensitivity to paramagnetic effects compared to lower field strengths (Spincemaille et al., 2020; Feinberg et al., 2023). Yet, physiologically induced magnetic field inhomogeneities stemming from tissue displacement during image encoding, e.g., arm motion, swallowing, and breathing scale with the main static magnetic field strength and become particularly prominent at field strengths above 3 T. To compensate for this, in this study real-time dynamic shimming continuously compensates for subject-and system-induced B₀ drifts during 3D GRE sequences, resulting in significantly improved image quality at high resolution as described earlier (Duerst et al., 2016). Regarding the acquisition of GRE images, the here applied single-echo technique could be seen as a potential limitation as multiple-echoes lead to a better signal to noise ratio (Gharabaghi et al., 2020). The fact, that images were acquired with one single echo is owed to the fact that real-time field control setup was used which is not compatible with multi-echoes. Yet, single-echo also simplifies phase unwrapping by avoiding multi-echo phase concatenation. In our experience with this technique, the reduction of physiologically induced field inhomogeneities resulting in image artifacts through real-time field control outweighs the benefits of multi-echoes in terms of image quality (Duerst et al., 2016).

To our knowledge, our study is the first to apply QSM imaging with UHF MRI at 7 T combined with real-time field control to investigate a clinical population. Our findings may encourage further use of this experimental approach for significantly enhancing image quality of high-resolution MRI of regionally confined and small brain structures, as well as implementation in commercially available systems.

A notable limitation of the present study is the small sample size, as well as the cross-sectional design of the current study. The here first used approach of combining UHF MRI at 7 T with real-time field cameras for investigating a clinical study population resulted in significantly increased image quality at high resolutions (De Zanche et al., 2008; Duerst et al., 2015; Duerst et al., 2016). However, the effort associated with establishing the experimental setup limited the size of the study population, as well as longitudinal follow-up. However, the increase in measurement precision by increased image-quality of the here used setup might increase power of our approach, and as such might compensate for the rather small sample size.

In conclusion, our data suggest that aging related cognitive dysfunction may be associated with distinct patterns of iron distribution in the EC-hippocampus system. Future longitudinal studies are needed to confirm whether iron deposition patterns might serve as complementary biomarkers of non-AD co-pathology (Jack et al., 2024), as well as their integration with blood biomarkers (Ashton et al., 2024) for an individual assessment of aging related cognitive disorder.

## Data Availability

The raw data supporting the conclusions of this article will be made available by the authors, without undue reservation.

## Footnotes

^1^https://surfer.nmr.mgh.harvard.edu/

## Glossary

Aβ: Amyloid beta
AD: Alzheimer’s disease
APOE: Apolipoprotein E
BNT: Boston naming test
EC: Entorhinal cortex
FOV: Field of view
GRE: Gradient recalled echo
HC: Healthy control
MCI: Mild cognitive impairment
MMSE: Mini-mental state examination
MP2RAGE: Magnetization-prepared 2 rapid acquisition gradient echoes
MRI: Magnet resonance imaging
NMR: Nuclear magnetic resonance
QSM: Quantitative susceptibility mapping
T: Tesla
TE: Echo time
TI: Inversion time
TR: Repetition time
TMT: Trail making test
TSE: Turbo spin echo
UHF: Ultra-high field
VLMT: Verbal learning and memory test
